# Identifying Terminologies Used Prior to the Onset of Interstitial Lung Disease in Patients With Lung Cancer: Descriptive Analysis of Electronic Medical Record Data

**DOI:** 10.2196/70603

**Published:** 2025-11-03

**Authors:** Masami Mukai, Hiroki Adachi, Tomohiro Yamaguchi, Ryunosuke Tanabe, Yasuo Sugitani, Yoshimasa Hanada, Noriaki Nakajima, Naoki Mihara

**Affiliations:** 1 Division of Medical Informatics, National Cancer Center Hospital, 5-1-1 Tsukiji, Chuo-ku, Tokyo, 104-0045, Japan, 81 335422511; 2 Biometrics Department, Chugai Pharmaceutical Co., Ltd., Tokyo, Japan; 3 Safety Science 1 Department, Chugai Pharmaceutical Co., Ltd., Tokyo, Japan; 4 Oncology Lifecycle Management Department, Chugai Pharmaceutical Co., Ltd., Tokyo, Japan; 5 Department of Medical Informatics, Jinsenkai MI Clinic, Toyonaka, Osaka, Japan

**Keywords:** electronic health record, electronic medical record, interstitial lung disease, interstitial pneumonia, lung cancer, lung neoplasms, natural language processing

## Abstract

**Background:**

The growing importance of real-world data (RWD) as a source of evidence for drug effects has led to increased interest in clinical research utilizing secondary use data from electronic medical record systems. Although immune checkpoint inhibitors and targeted therapies have advanced lung cancer treatment, managing complications such as interstitial lung disease (ILD) remains challenging. Early detection and prevention of ILD are crucial for improving patient prognosis and quality of life; however, predictive biomarkers have yet to be established. Therefore, methods to identify ILD risk factors and enable early detection using RWD are needed.

**Objective:**

This exploratory study aimed to identify associated factors and prodromal symptoms of ILD onset using clinical data stored in a hospital information system.

**Methods:**

Clinical data of patients diagnosed with stage IV lung cancer between November 2011 and December 2018 were extracted from the hospital information system of the National Cancer Center Hospital in Japan. A total of 3 patient groups were defined: the ILD Set, based on laboratory test results and radiological records; the ILD-GC Set, which added glucocorticoid treatment to the ILD Set; and the No ILD Set, for patients without ILD. The primary endpoint was the frequency of Japanese words extracted from electronic medical records, specifically from notes in the Problem-Oriented System/Subjective, Objective, Assessment and Plan format. Noun frequencies were compared between the ILD or ILD-GC Sets and the No ILD Set. Free-text data were processed using morphological analysis, and terms were categorized using the Patient Disease Expression Dictionary or the World Health Organization Drug Dictionary. Key terms were extracted from physician and nurse records based on the descending order of ranking differences to identify associated factors and prodromal symptoms.

**Results:**

The analysis included 674 cases (105 in the ILD Set [including 12 in the ILD-GC Set] and 569 in the No ILD Set). Baseline characteristics showed no apparent differences across groups. In the 30 days prior to ILD onset, notable differences in word frequencies per 1000 notes between the ILD-GC Set and No ILD Set were observed in the following term categories: respiratory symptoms (eg, breathlessness, shortness of breath, oxygen), ranging from 170.59 to 46.51; pain or analgesics (eg, Lyrica [pregabalin], soreness, precordial pain, opioids), ranging from 462.88 to 45.16; and appetite-related terms (eg, inappetence, food intake, queasiness, Novamin [prochlorperazine]), ranging from 102.23 to 51.90.

**Conclusions:**

Terms related to respiratory symptoms, pain or analgesics, and appetite were identified as associated factors for ILD onset in patients with stage IV lung cancer using RWD from acute care institutions for malignant tumors. These findings may support the early detection of ILD and underscore the potential of RWD to generate real-world evidence that informs drug discovery and pharmaceutical development.

## Introduction

Since the late 2010s, pharmaceutical regulatory authorities, such as the US Food and Drug Administration and European Medicines Agency, have referred to medical data routinely collected in health care institutions and a variety of sources as real-world data (RWD) [1,2]. They began to examine the usefulness of RWD as a source of evidence for various drug effects. In recent years, the importance of conducting clinical research to obtain new insights by reusing diagnostic information stored in hospital information systems, primarily electronic medical record (EMR) systems, as a source of data has increased [3]. EMR data can be broadly categorized into structured and unstructured formats. Unstructured data include electronic health records, free-text narratives, and test reports (eg, radiation reports), which often contain key clinical details such as patient symptoms, treatment intent, and symptom outcomes. Information on the effectiveness and safety of cancer treatments is typically embedded in these unstructured data sources. However, the unstructured nature of these records presents challenges for the immediate and accurate extraction of relevant information. Natural language processing techniques offer potential for extracting symptom-related data from unstructured EHR free-text narratives [4]. Furthermore, integrating structured and unstructured data (eg, medical records and radiological reports) may enhance the evaluation of safety information and aid in identifying initial symptoms or other critical clinical information [5].

Lung cancer is reported to have the highest incidence and mortality among various cancer types worldwide [6]. The treatment strategy for lung cancer depends on the histological subtype, stage, and oncogenic driver mutations or alterations; systematic treatment (ie, medication) is recommended for advanced or metastatic lung cancer [7-12]; its efficacy has been improved by immune checkpoint inhibitors [13-18], and personalized medicine for lung cancer has advanced with the advent of targeted therapies for driver mutations and companion diagnostics [19-25] in the past decade. However, these treatments have been reported to induce interstitial lung disease (ILD) as a toxic effect in 1.6%‐5% patients [26,27]. ILD not only makes it difficult to continue treatment but also results in respiratory failure or death, especially in patients with pulmonary fibrosis [28]. Hence, establishing risk factors and prodromal symptoms is crucial. Patient selection or early intervention can help prevent the worsening of ILD, thereby contributing to improved patient prognosis and quality of life. Although Krebs von den Lungen-6 (KL-6) [29] and surfactant protein D (SP-D) [30] are important diagnostic biomarkers of ILD, there is no established biomarker for predicting ILD onset [31,32]. Additionally, conducting diagnostic examinations, such as blood tests or diagnostic modalities like computed tomography, frequently for the sole purpose of ILD risk assessment is not reasonable in terms of cost-effectiveness or radiation exposure. Age, preexisting lung disease, preexisting ILD, idiopathic pulmonary fibrosis, smoking, drug dosage, and poor performance status have been reported as risk factors for drug-induced ILD in systematic reviews [33]; however, time-dependent risk factors have not been sufficiently investigated. Another study has reported the initiation of clinical trials to construct onset-prediction models using wearable devices [34]; nevertheless, a solid model has not yet been established. Thus, a novel time-dependent approach is warranted for information on ILD onset, and extracting symptoms from free-text narratives of electronic health records [4] can provide insights into ILD development in real-world settings; to the best of our knowledge, such an approach has not been previously reported. Therefore, we conducted an exploratory study using the EMR in a hospital information system to identify associated factors and prodromal symptoms of ILD onset, mainly focusing on the preceding period of ILD development.

## Methods

### Study Design

This was an exploratory observational study that used anonymized medical records from the National Cancer Center Hospital in Japan for secondary use. Patients diagnosed with stage IV lung cancer between November 2011 and December 2018 who provided comprehensive informed consent regarding registration in the biobank [35] were eligible for the study. We excluded patients who were enrolled in clinical trials between November 2011 and December 2019, those who had a disease that fewer than 10 patients had in the anonymized database, and those who explicitly refused to participate in the study after being informed about the study’s details and their option to opt-out (Textbox 1).

Textbox 1. **Inclusion and exclusion criteria and definition of patient sets.**

**Inclusion criteria**

 Diagnosis of Stage IV lung cancer from November 2011 to December 2018 (to enable a 1-year follow-up period before the end of the extraction period)

 Comprehensive informed consent for the biobank

**Exclusion criteria**

 Enrollment in clinical trials from November 2011 to December 2019

 Disease that fewer than 10 patients had in the anonymized database

 Patients explicitly refused to participate in the study based on the contents of the study disclosed for opt-out

**Patient set definition**

*Common algorithm*

 ICD10 code of C34 (malignant neoplasm of bronchus and lung)

 Exclude patients with any of the following documentation within 30 days from the first visit

  Abnormal laboratory test result (KL-6>500 U mL^–1^ or surfactant protein D [SP-D]>110 ng mL^–1^

  Interstitial lung disease (ILD)-related documentation (diagnosis of ILD or ILD-related irregular findings in radiation reports)

*ILD Set*

 Patients with any of the following documentation after 30 days from the first visit.

  Abnormal laboratory test result (KL-6>500 U mL^–1^ or SP-D>110 ng mL^–1^)

  ILD-related documentation (diagnosis of ILD or ILD-related irregular findings in radiation reports)

*ILD With the Glucocorticoid Treatment Set*

 Patients with all of the following documentation:

  All of the following documentation within 7 days

   Abnormal laboratory test result (KL-6>500 U mL^–1^ or SP-D>110 ng mL^–1^)

   ILD-related documentation (diagnosis of ILD or ILD-related irregular findings in radiation reports)

  Glucocorticoid therapy (including steroid pulse therapy) within 7 days after the ILD onset

*No ILD Set*

 Patients without any of the following documentation:

  Abnormal laboratory test result (KL-6>500 U mL^–1^ or SP-D>110 ng mL^–1^)

   ILD-related documentation (diagnosis of ILD or ILD-related irregular findings in radiation reports)

### Data Collection

Patients diagnosed with stage IV lung cancer until December 2018 who provided comprehensive informed consent for biobank participation were included. Data from November 2011 to December 2019 were extracted. Algorithms to identify the ILDs based on the radiation reports and laboratory test results were used because identification using only recorded disease names may result in the misidentification of diagnosis and its development date (eg, it may include ILD initially developed in former hospitals or differences between the development date and the diagnosis date). ICD-10 code C34 was used to identify patients with lung cancer. The ILD Set, ILD with Glucocorticoid Treatment Set (ILD-GC Set), and No ILD Set were defined using the following algorithms. Patients with documentation of an abnormal laboratory test result or ILD-related documentation in the radiation reports within 30 days of the first visit were excluded from each analysis set because the duration was insufficient to evaluate early symptoms. The ILD Set was defined as patients with an abnormal laboratory test result (KL-6>500 U mL^–1^ or SP-D>110 ng mL^–1^), or ILD-related documentation (diagnosis of ILD or ILD-related abnormal findings) in the radiation reports 30 days after the first visit. To focus on patients who reliably developed ILDs, the ILD-GC Set was defined as patients with an abnormal laboratory test result and ILD-related documentation in the radiation reports within 7 days and who received a glucocorticoid therapy (including steroid pulse therapy) within 7 days after the onset of ILD. Glucocorticoid therapies used as supportive care for anticancer therapy were excluded from the analysis. The No ILD Set was defined as patients without an abnormal laboratory test result or ILD-related documentation in the radiation reports. The ILD onset date was defined as the earliest date of documented abnormal laboratory test results or ILD-related documentation in the radiation reports in the ILD Set, and the earliest date of documented abnormal laboratory test results and ILD-related documentation in the radiation reports within 7 days in the ILD-GC Set. ILD-related findings were documented as follows: interstitial pneumonia, traction bronchiectasis, reticular abnormalities, diffuse involvement, and ground-glass opacity [36].

### Outcome Measures

**Figure 1.**
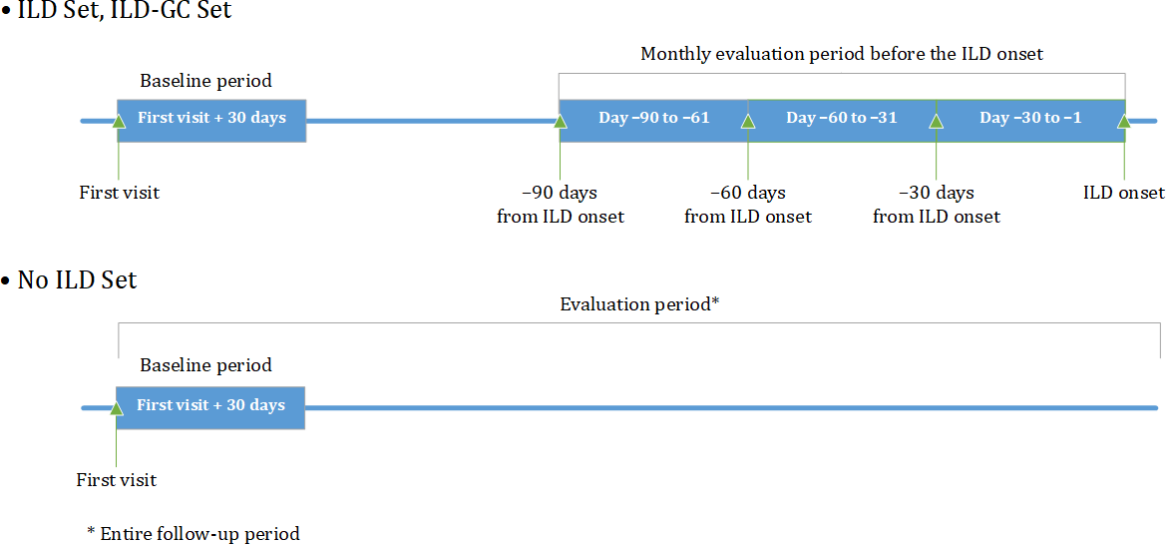
Baseline and pre-interstitial lung disease (ILD) onset periods. ILD-GC: ILD with Glucocorticoid Treatment Set.

The primary endpoint was the frequency of words in the medical charts in Japanese. Word frequencies were calculated by writers (physicians or nurses), time windows, and patient sets. Only nouns from notes in the Problem, Subjective, Objective, or Assessment sections of the Problem-Oriented System/Subjective, Objective, Assessment, and Plan format were included in the frequency calculation (the Plan section was not used). The difference in word frequencies between the ILD, ILD-GC, and No ILD Sets was calculated to evaluate the distinctive words seen in patients who developed ILD. Age, sex, height, and weight were collected as baseline characteristics. To evaluate the potential detection ability and presentation timing of irregular values for ILD, temporal trends of laboratory test results (white blood cell count, platelet count, neutrophil count or percent, hemoglobin, alanine transaminase, aspartate transaminase, serum bilirubin, gamma-glutamyl transferase, serum creatinine, free thyroxine, thyrotropin, KL-6, SP-D, and C-reactive protein [CRP] levels) were evaluated using time windows. The laboratory results closest to ILD onset or the date of the first visit were used as representative values. To investigate drugs potentially associated with ILD induction, the temporal administration status of anticancer medications was described using time windows before ILD onset. The time windows were divided into 2 categories: baseline (background information from the first visit date to 30 d after) and monthly intervals before the onset of ILD (days –1 to –30, –31 to –60, and –61 to –90). These intervals were based on the typical 3‐ to 4-week cycle of lung cancer regimens. The frequency of words in the No ILD Set was calculated during the entire follow-up period as a control that reflected the trend, regardless of specific periods, after the first visit date (Figure 1).

### Data Processing

The EMR database from the National Cancer Center, Japan, including information on medical charts, radiation reports, medications, and laboratory test results, was anonymized before analyses by using rule-based processing with regular expressions. These anonymization techniques were applied to mask patient identifiers, names, facility information, addresses, and phone numbers, following methodologies described previously [37,38]. Because data were generated from a single hospital, there was no linkage. Because there is insufficient research on associated factors and prodromal symptoms of ILD onset, we began by analyzing traditional word frequencies to understand the actual situation.

No large language models (LLMs) were used in this study. While LLMs have recently been utilized for the extraction of data, such as that on cancer progression events from EMR [39], to our knowledge, there are no established models suitable for the task aligned with this study’s objective of exploring a wide range of undefined prodromal symptoms and risk factors for ILD. Furthermore, traditional word-frequency analysis is based on the direct tabulation of word occurrences, providing transparency in the derivation process and allowing for an intuitive interpretation of results. Because Japanese is an unsegmented language (ie, words are not separated with spaces or other letters in sentences), free text from medical charts was processed using the morphological analysis method to extract words related to symptoms or treatments. This natural language processing approach produces segmented words from non-space-delimited sentences, which was performed using a Japanese morphological analysis tool, Mecab (version 0.966 [32 bit]; Taku Kudo) [40]. ComeJisyo (version Utf8-3) [41], a Japanese practical medical terminology dictionary, was specified as the user dictionary for Mecab to enable the extraction of words with consideration for medical information. Nouns from the output results of Mecab’s morphological analysis were treated as target words for aggregation. Words with no medical meaning or interpretation (eg, symbols, numbers, or units) were excluded. As sentences in past notes were copied and reused repeatedly, the same descriptions in the same patient’s past notes were excluded by sentence unit to analyze only newly added descriptions.

The words with a larger difference in frequency in the ILD or ILD-GC Set were classified into term categories in a post hoc manner to facilitate interpretation, to correct for fluctuations in descriptions, and to organize words with the same meaning or background. The words related to symptoms were classified with reference to the Patient Expression Dictionary [42] and clinical perspective. The words related to medicine for the same treatment purpose were classified with reference to the standardized drug groupings in the WHO Drug Dictionary [43]. The categories of pain-related and analgesic medications were defined as N02 and M02 of the ATC classifications in the WHO Drug Dictionary, respectively. At least 1 author implemented the classification of words into the categories, and other authors reviewed and confirmed its validity. This classification work was conducted entirely in Japanese and subsequently translated into English. For the English translation of symptoms, we utilized Medical Dictionary for Regulatory Activities (MedDRA) version 27.1, a medical terminology dictionary developed by the International Council for Harmonisation of Technical Requirements for Pharmaceuticals for Human Use [44]. For pharmaceutical product names (brand names), we employed the English names as documented in the package inserts, while generic drug names were directly translated into their English equivalents.

### Ethical Considerations

This study was conducted in accordance with the Ethical Guidelines for Medical and Health Research Involving Human Subjects [45,46]. This study was approved by the ethics committee registered with the Ministry of Health, Labour and Welfare (Registration No. 11000489). The approval of the ethical committee covered this secondary analysis study without the requirement for additional consent [1]. The opportunity to opt out was provided to patients, along with publicly disclosed information about the research implementation, in accordance with guidelines. This approach was taken as this study did not involve invasive procedures or interventions and used only information, including the results of examinations that already existed prior to the preparation of the research protocol. The disclosed information included the significance, purpose, and methodology of the research; the name of the research institution; and contact information for inquiries and complaints. This study utilized anonymized medical records from the National Cancer Center Hospital, and patient anonymity and confidentiality were strictly maintained. No compensation was provided, as this study was a secondary analysis utilizing existing data. The data are not publicly available, and approvals are required to access the EHR database.

### Statistical Analysis

As this exploratory study used an existing database for secondary use, sample size calculations based on formal statistical tests were not implemented. We included all registered patients who met the eligibility criteria in the existing database. Word frequencies were calculated as the total appearance per 1000 notes. The difference in frequency in the ILD or ILD-GC Sets compared to the No ILD Set was described (the specific calculation formula is presented in Supplementary Material 1 in Multimedia Appendix 1).

Descriptive analyses of continuous variables were performed using the mean, median, standard deviation, minimum, 25th percentile, 75th percentile, and maximum. Imputation methods for missing values were not implemented. The results of laboratory tests were aggregated without missing data. Word frequencies were counted if the word existed in the notes. All statistical analyses were performed using Python (version 3.7.6; Python Software Foundation) or R (version 3.6.1; R Foundation for Statistical Computing).

## Results

### Participants

**Figure 2.**
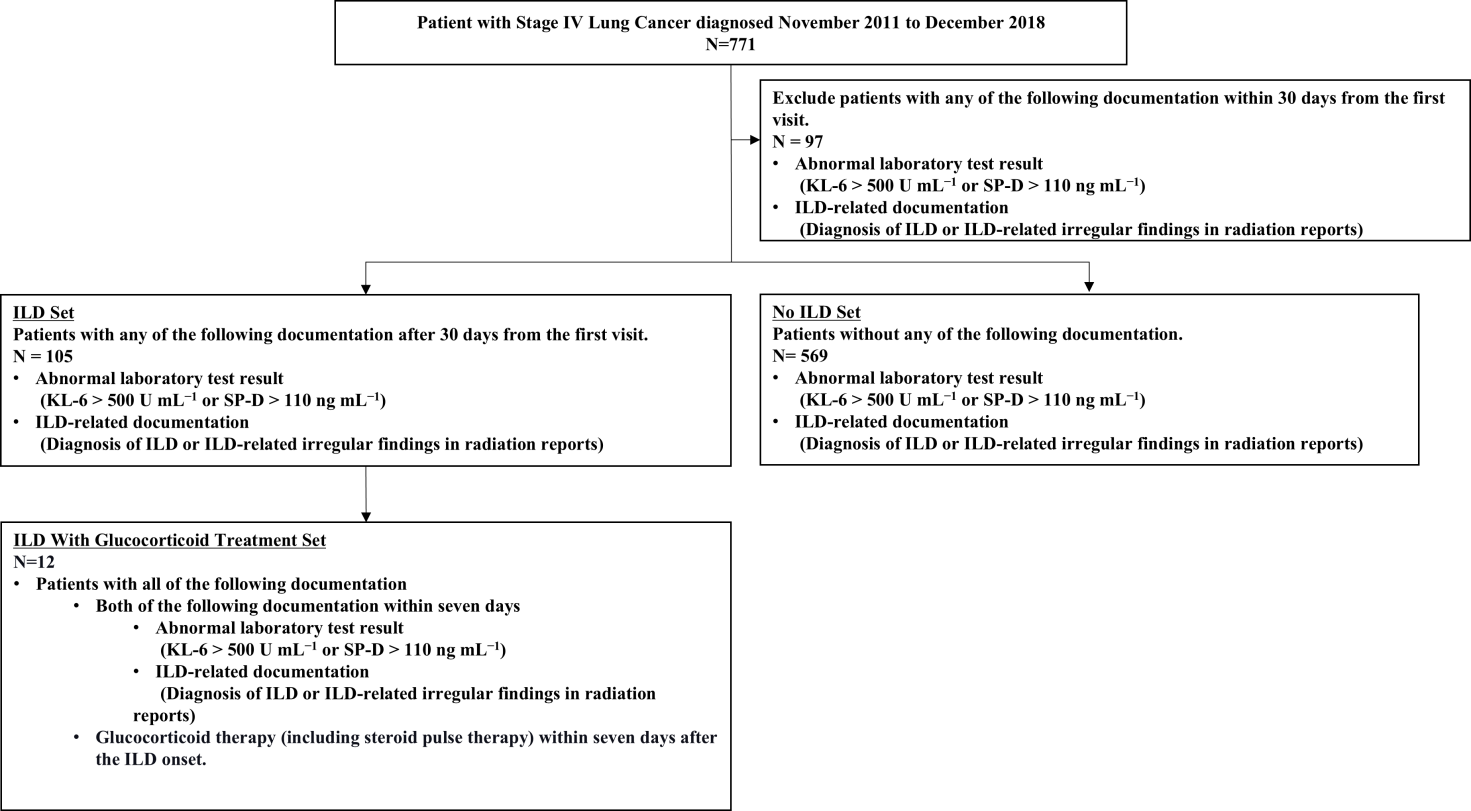
Flow diagram of analysis sets. ILD: interstitial lung disease; KL-6: Krebs von den Lungen-6; SP-D: surfactant protein D.

Between November 2011 and December 2018, 771 patients were diagnosed with stage IV lung cancer and met the inclusion criteria. Of these, 97 patients had abnormal laboratory test results or ILD-related documentation in the radiation reports within 30 days of their first visit and were excluded from the analysis set. The ILD, ILD-GC, and No ILD Sets included 105, 12, and 569 patients, respectively (Figure 2).

### Descriptive Data Analysis

#### Baseline Characteristics

There were no apparent differences between the ILD, ILD-GC, and No ILD Sets. The mean age (SD) was 62.8 (11.7), 66.6 (7.4), and 65.2 (12.3) years, respectively. The proportions of male patients were as follows: 60.0% (63/105) in the ILD Set, 66.7% (8/12) in the ILD-GC Set, and 61.2% (348/569) in the No ILD Set (Table 1).

**Table 1.** Baseline characteristics of the patients.

Characteristic	ILD Set (N=105)	ILD-GC Set^a^ (N=12)	No ILD^b^ Set (N=569)
Age, years, mean (SD)	62.8 (11.7)	66.6 (7.4)	65.2 (12.3)
Sex, n (%)			
Male	63 (60.0)	8 (66.7)	348 (61.2)
Female	42 (40.0)	4 (33.3)	221 (38.8)
Height, cm, mean (SD)	161.8 (8.0)	162.1 (9.0)	162.1 (11.8)
Weight, kg, mean (SD)	55.7 (14.4)	55.7 (23.7)	55.1 (11.6)

a ILD-GC Set: ILD With Glucocorticoid Treatment Set.

b ILD: interstitial lung disease.

#### Frequency of Words in the Medical Charts in Japanese

To understand the trend of symptoms recorded before ILD onset, we focused on words with higher word frequencies in the ILD and ILD-GC Sets compared to non-ILD cases immediately before ILD onset (days –30 to –1). Words recorded for only 1 patient were excluded as they might have been used specifically for the patient. Descriptions related to units or alphanumeric characters without a clear medical significance were also excluded.

Tables2  3 summarize the top 50 words with the highest word frequencies by physicians and nurses, respectively, in the ILD-GC Set immediately before ILD onset (days –30 to –1). Words with negative frequency values appear less frequently in the ILD-GC Set or ILD Set than in the No-ILD Set. The words were classified into term categories to facilitate interpretation (Table 4).

**Table 2.** The differences in the frequency of words written by physicians between the interstitial lung disease with Glucocorticoid Treatment (ILD-GC) and No ILD Sets.

Word	Difference in the frequency of words in –30 to –1 day(s)	Number of patients for which the words were recorded in –30 to –1 day(s)	Difference in the frequency of words in –31 to –60 day(s)	Difference in the frequency of words in –61 to –90 day(s)	Term category
Lyrica (product name of pregabalin)	209.76	3	15.88	84.76	Pain or analgesic-related
Pain	175.64	8	248.13	34.23	Pain or analgesic-related
Sleepiness	170.88	2	85.22	−15.99	Sleepiness
Breathlessness	170.59	2	32.30	11.50	Respiratory symptom-related
Yesterday	160.57	8	52.75	48.2	—^a^
Improvement	158.11	8	65.44	33.11	—
Morning	145.65	5	71.10	57.26	—
AM	134.96	4	65.50	198.09	—
Soreness	119.83	7	161.68	−15.27	Pain or analgesic-related
Restart	114.00	5	101.10	−12.26	—
Reduction	110.68	3	11.76	−20.63	—
Degree	104.46	4	32.88	−18.01	—
After	91.46	6	125.14	10.66	—
Worsening	89.73	5	39.39	0.09	—
Meal	87.56	5	−8.31	62.31	—
Anxiety	86.60	2	6.83	−9.36	—
NRS^b^	84.25	4	54.11	42.58	Pain or analgesic-related
Anemia	83.67	2	−8.24	−12.29	Anemia
This-day	75.42	6	49.45	0.92	—
Today	73.83	4	59.89	18.28	—
Oxycodone	68.81	3	120.60	219.06	Pain or analgesic-related
Tomorrow	67.83	5	3.26	22.38	—
Okay	67.44	3	34.25	3.05	—
Night	66.94	2	45.86	22.75	—
Right-chest	66.41	4	28.09	23.48	—
Decrease	61.03	6	47.04	−15.99	—
Exacerbation	58.88	3	47.94	−23.19	—
Delirium	58.83	2	47.85	−16.93	Delirium
Bleeding	58.67	2	−3.94	−12.04	Bleeding
Discontinuation	58.47	3	42.44	5.44	—
Bleeding-source	55.19	2	−0.37	−0.37	—
Skin-eruption	54.42	3	−15.25	10.23	Skin-eruption
Increase	54.27	3	25.09	148.97	—
Use	54.17	5	62.43	−12.75	—
Test/Trial	50.15	2	−1.36	−5.41	—
Condition	48.93	3	13.66	0.95	—
Cause	48.90	3	19.68	66.57	—
Response	48.85	3	−11.72	51.37	—
Ache	48.46	3	36.43	1.74	Pain or analgesic-related
O_2_	46.51	7	−12.77	93.23	Respiratory symptom-related
Consideration	45.82	7	89.51	57.18	—
Conversation	45.70	2	19.48	22.97	—
Last night	45.64	3	15.37	−4.87	—
Rescue	45.37	2	56.63	63.04	—
OxyContin (product name of oxycodone)	45.16	3	8.84	26.22	Pain or analgesic-related
Awareness	45.03	3	25.91	3.36	—
Nasal	43.96	2	13.69	−6.55	—
Face	43.96	2	−2.50	7.34	—
Drain	43.51	2	69.92	−7.00	—
Residual	43.31	4	41.38	6.69	—

a —: not applicable.

b Due to ambiguity caused by word segmentation, some terms, eg, NRS, cannot be clearly defined.

**Table 3.** The differences in the frequency of words written by nurses between the ILD-GC and No ILD Sets.

Word	Difference in the frequency of words in –30 to –1 day(s)	Number of patients for which the words were recorded in –30 to –1 day(s)	Difference in the frequency of words in –31 to –60 day(s)	Difference in the frequency of words in –61 to –90 day(s)	Term category
Pain	462.88	7	300.64	785.96	Pain or analgesic-related
OxyContin (product name of oxycodone)	236.45	4	219.67	5.68	Pain or analgesic-related
NRS^b^	235.03	7	144.12	65.80	Pain or analgesic-related
Oxinorm (product name of oxycodone)	216.78	5	187.41	647.55	Pain or analgesic-related
Oral administration	191.12	7	135.17	237.27	—^a^
Impact	169.59	5	235.32	138.82	—
Opioid	153.21	5	44.12	68.59	Pain or analgesic-related
Most	149.16	3	30.28	−20.07	—
Worry	142.06	2	−24.38	−42.56	—
Breathlessness	130.25	4	23.96	30.25	Respiratory symptom-related
Increase	123.99	3	63.85	108.61	—
Course	122.68	6	3.80	222.68	—
This-day	111.10	9	22.99	−150.43	—
Inappetence	102.23	3	−18.05	−36.23	Appetite-related
Soreness	99.03	8	177.35	245.18	Pain or analgesic-related
Drug-name	92.81	2	117.99	−45.65	—
Usage	91.84	2	117.02	−8.16	—
Right-back	88.74	3	14.61	34.89	—
AM	88.31	2	110.68	272.92	—
Possibility	88.00	4	−63.05	−81.23	—
Administration	85.01	3	37.46	−53.45	—
Anxiety	84.18	3	−30.5	−85.05	—
During movement	83.96	4	18.23	−54.50	—
Novamin (product name of prochlorperazine)	83.81	3	−5.70	14.58	Appetite-related
Family	78.32	2	−48.95	−6.30	—
Timing	74.93	2	−17.38	21.08	—
Shortness of breath	74.85	3	55.27	21.00	Respiratory symptom-related
Fall	72.24	6	62.45	56.86	—
Doctor	69.47	4	164.57	100.24	—
Start	68.10	4	−10.22	14.25	—
Watchful-waiting	64.09	4	−12.83	25.63	—
OxyContin-increased	60.16	2	−1.38	−1.38	Pain or analgesic-related
Face-scale	60.16	2	16.80	306.31	—
Pattern	59.21	2	18.65	20.75	—
Relief	59.03	4	78.61	66.72	—
Pain-precordial	58.94	3	33.76	74.32	Pain or analgesic-related
Fall-risk	57.34	6	−0.01	26.57	—
Going-out	56.94	2	34.57	210.79	—
Today	56.58	4	−9.15	−81.88	—
Medical-condition	55.68	2	36.10	1.83	—
Feeling-queasy	54.85	2	95.41	47.15	Appetite-related
Before	54.79	3	79.97	147.10	—
Sp^b^	54.26	3	104.61	169.65	—
Hgb^b^	54.09	3	−3.26	−130.53	—
Oxycodone	53.06	2	202.71	314.60	Pain or analgesic-related
O_2_	52.71	3	103.06	168.10	Respiratory symptom-related
Food-intake	51.90	3	−25.02	−25.02	Appetite-related
Intermittent	50.98	2	25.80	27.90	—
Discontinuation	50.52	2	9.96	12.06	—
Average	49.92	2	−11.62	26.84	—

a —: not applicable.

b Due to ambiguity caused by word segmentation, some terms, eg, NRS, Sp, and Hgb, cannot be clearly defined.

**Table 4.** Term categories in the interstitial lung disease with Glucocorticoid Treatment Set (ILD-GC Set).

Term categories	Word
Respiratory symptom related	Breathlessness, Shortness of breath, O_2_
Pain or analgesic related	Soreness, Ache, Pain, Pain-precordial, NRS^a^, Lyrica (product name of pregabalin), Opioid, Oxinorm (product name of oxycodone), OxyContin (product name of oxycodone), OxyContin-increased, Oxycodone
Appetite related	Inappetence, Food-intake, Feeling-queasy, Novamin (product name of prochlorperazine)
Delirium	Delirium
Anemia	Anemia
Bleeding	Bleeding
Sleepiness	Sleepiness
Skin eruption	Skin eruption
Others	AM, Hgb, nasal, Sp, Drain, Pattern, Face-scale, Rescue, Worsening, Most, Right-chest, Right-back, Impact, Possibility, Family, Conversation, Improvement, Start, Going-out, Intermittent, Face, Course, Watchful-waiting, Relief, Consideration, Cause, Reduction, After, Today, Restart, Yesterday, Last night, Residual, Use, Usage, Test/Trial, Timing, Awareness, Bleeding source, Meal, Worry, Doctor, Before, Exacerbation, Increase, During movement, Response, Okay, Discontinuation, Morning, Condition, Decrease, Degree, Fall, Fall-risk, Administration, Oral administration, Medical condition, Anxiety, Average, This day, Tomorrow, Night, Drug name

a Due to ambiguity caused by word segmentation, some terms, eg, NRS, Sp, and Hgb, cannot be clearly defined.

Among the words with increased word frequencies by physicians in the ILD-GC Set immediately before ILD onset (days –30 to –1), those related to symptoms or medication names were Lyrica (product name of pregabalin), Pain, Sleepiness, Breathlessness, Soreness, Anxiety, NRS, Anemia, Oxycodone, Delirium, Bleeding, Skin eruption, Ache, O_2_, and OxyContin (product name of oxycodone).

Among the words with increased word frequencies by nurses in the ILD-GC Set immediately before ILD onset (days –30 to –1), those related to symptoms or medication names were Pain, OxyContin (product name of oxycodone), NRS, Oxinorm (product name of oxycodone), Opioid, Breathlessness, Inappetence, Soreness, Anxiety, Novamin (product name of prochlorperazine), Shortness of breath, OxyContin-increased, Pain-precordial, Feeling queasy, Oxycodone, O_2_, and Food-intake.

In the ILD Set, similar to the ILD-GC Set, words related to symptoms or medication names with increased word frequencies by physicians immediately before ILD onset (days −30 to −1) were as follows: O_2_, Lyrica (product name of pregabalin), Pain, Sleepiness, Breathlessness, Soreness, NRS, Anemia, Oxycodone, and Delirium. Other words related to symptoms or medication names with increased word frequencies included Fever, BP, RT, Tenderness, Opso (product name of morphine), ERCP, Stomatitis, Metastasis, and Exacerbation (Table S1 in Multimedia Appendix 1).

In the ILD Set, similar to that in the ILD-GC Set, words related to symptoms or medication names with increased word frequencies by nurses immediately before ILD onset (days -30 to -1) were OxyContin (product name of oxycodone), NRS, Opioid, Breathlessness, Soreness, Novamin (product name of prochlorperazine), Shortness of breath, and Oxycodone. Other words related to symptoms or medication names with increased word frequencies were Stomatitis, Fever, Sleepiness, Edema, Pneumonia, Right precordial, Dizziness/Vertigo, and Dyspnea (Table S2 in Multimedia Appendix 1).

In the ILD-GC Set, for words related to symptoms or medication names that showed increased word frequencies by physicians immediately before ILD onset (days –30 to –1), the number of patients with these words documented increased (Table S3 in Multimedia Appendix 1). Similarly, for words related to symptoms or medication names that showed increased word frequencies by nurses immediately before ILD onset (days –30 to –1), the number of patients with these words documented increased (Table S4 in Multimedia Appendix 1). As with the ILD-GC Set, in the ILD Set, words related to symptoms or medication names that showed increased word frequencies by physicians or nurses immediately before ILD onset (days –30 to –1) also showed an increase in the number of patients with these words documented (Tables S5 and S6 in Multimedia Appendix 1). A corresponding table of terms used in the Japanese medical charts and their English translations is presented as Table S7 in Multimedia Appendix 1.

#### Term Categories and Their Constituent Words

We focused on the top 50 words with higher word frequencies by physicians or nurses in the ILD-GC Set compared to the non-ILD Set during the period immediately preceding ILD onset (days –1 to –30) (Table 4).

#### Temporal Trends in the Laboratory Test Results

Trends in CRP, KL-6, and SP-D levels, which are known to be particularly elevated with the onset of ILD, are shown in Table 5. No other clinical laboratory tests showed explicit changes before ILD onset.

In both ILD-GC and ILD Sets, the mean CRP values increased toward the date of ILD onset. However, in the ILD Set, the median remained at 0.9 from days −30 to −1, confirming that the CRP levels did not clearly increase in more than half of the patients. In contrast, in the ILD-GC Set, the median increased to 8.2 from days −30 to −1.

**Table 5.** Temporal trends in the laboratory test results.

Laboratory test results	ILD^a^ set (N=105)				ILD-GC Set^b^ (N=12)			
	Baseline	−90 to −61 day(s)^c^	−60 to −31 day(s)^c^	−30 to −1 day(s)^c^	Baseline	−90 to −61 day(s)^c^	−60 to −31 day(s)^c^	−30 to −1 day(s)^c^
CRP^d^ (mg dL^−1^)								
n^e^ (%)	102 (97.1)	74 (70.5)	97 (92.4)	90 (85.7)	12 (100)	10 (83.3)	12 (100)	11 (91.7)
Mean	1.8	2.0	2.4	4.1	1.5	1.8	2.8	8.8
SD	2.8	3.5	4.3	6.1	2.1	2.3	3.1	6.6
Median	0.4	0.5	0.6	0.9	0.5	1.3	0.9	8.2
KL-6^g^ (ng mL^−1^)								
n^e^ (%)	4 (3.8)	1 (1.0)	5 (4.8)	3 (2.9)	0 (0)	0 (0)	0 (0)	0 (0)
Mean	293.5	481.0	281.4	245.0	—^f^	—	—	—
SD	95.6	—	89.3	43.6	—	—	—	—
Median	276.0	481.0	282.0	237.0	—	—	—	—
SP-D^h^ (U mL^−1^)								
n^e^ (%)	3 (2.9)	1 (1.0)	3 (2.9)	2 (1.9)	0 (0)	0 (0)	0 (0)	0 (0)
Mean	85.57	42.90	58.83	47.80	—	—	—	—
SD	28.12	—	46.50	16.26	—	—	—	—
Median	95.20	42.90	53.90	47.80	—	—	—	—

a ILD: interstitial lung disease.

b ILD-GC Set: ILD with Glucocorticoid Treatment Set.

c Monthly periods before the onset of ILD.

d CRP: C-reactive protein.

e Number of patients who had a laboratory test result.

f —: not applicable.

g KL-6: Krebs von den Lungen-6.

h SP-D: surfactant protein D.

#### KL-6 and SP-D

For both KL-6 and SP-D, only a few cases had test values measured consistently from days −90 to −61, −60 to −31, and −30 to −1, making it difficult to observe trends in the population. The aggregated values alone did not show any particular upward trend.

#### Administration Status of Anticancer Medications

The administration status of the anticancer medications is presented in Table 6. The period until ILD onset was divided into 30-day intervals, and the anticancer medications administered during each period were categorized by their mechanism of action and aggregated. If the same patient received different categories of anticancer medications during the same period, each category was counted as 1 case. If multiple anticancer medications were used concurrently within the same category, they were counted as 1 case.

**Table 6.** Temporal administration status of the anticancer medications.

Anticancer medication	ILD^a^ Set (N=105), n (%)			ILD-GC Set^b^ (N=12), n (%)		
	−90 to −61 day(s)^c^	−60 to −31 day(s)^c^	−30 to −1 day(s)^c^	−90 to −61 day(s)^c^	−60 to −31 day(s)^c^	−30 to −1 day(s)^c^
PD-1 inhibitors^d^	6 (5.7)	9 (8.6)	8 (7.6)	1 (8.3)	2 (16.7)	3 (25)
PD-L1 inhibitors^e^	0 (0)	1 (1.0%)	1 (1.0%)	0 (0)	0 (0)	0 (0)
EGFR-TKIs^f^	18 (17.1)	22 (21.0)	21 (20)	1 (8.3)	2 (16.7)	2 (16.7)
ALK-TKIs^g^	6 (5.7)	8 (7.6)	8 (7.6)	1 (8.3)	1 (8.3)	1 (8.3)
VEGF inhibitors^h^	2 (1.9)	3 (2.9)	4 (3.8)	0 (0)	0 (0)	0 (0)
Cytotoxic agents^i^	29 (27.6)	32 (30.5)	34 (32.4)	4 (33.3)	3 (25)	4 (33.3)
None	47 (44.8)	33 (31.4)	32 (30.5)	5 (41.7)	4 (33.3)	3 (25)

a ILD: interstitial lung disease.

b ILD-GC Set: ILD with Glucocorticoid Treatment Set.

c Monthly periods before the onset of ILD.

d Programmed cell death protein 1 inhibitors: pembrolizumab and nivolumab.

e Programmed death-ligand 1 inhibitors: atezolizumab.

f Epidermal growth factor receptor tyrosine kinase inhibitors: afatinib, osimertinib, gefitinib, and erlotinib.

g Anaplastic lymphoma kinase tyrosine kinase inhibitors: ceritinib, crizotinib, lorlatinib, and alectinib.

h Vascular endothelial growth factor inhibitors: bevacizumab.

i Cytotoxic agents: gemcitabine, amrubicin, docetaxel, paclitaxel, cisplatin, pemetrexed, irinotecan, carboplatin, and etoposide.

In the ILD-GC Set, the categories of anticancer medications administered from 30 days before to the day before ILD onset were as follows: programmed cell death protein 1 (PD-1) inhibitors (3 cases), epidermal growth factor receptor tyrosine kinase inhibitors (EGFR-TKIs) (2 cases), anaplastic lymphoma kinase tyrosine kinase inhibitors (ALK-TKIs) (1 case), and cytotoxic agents (4 cases). In total, 3 patients did not receive any anticancer medications. On the other hand, in the ILD Set, these categories were as follows: PD-1 inhibitors (8 cases), programmed death-ligand 1 inhibitors (1 case), EGFR-TKIs (21 cases), ALK-TKIs (8 cases), vascular endothelial growth factor inhibitors (4 cases), and cytotoxic agents (34 cases). A total of 32 patients did not receive any anticancer medications.

## Discussion

### Principal Results

We explored the associated factors and early symptoms of ILD using articles, records, and various examination reports stored in the hospital information system of a specialized hospital for acute care and treatment of malignant tumors. We used algorithms to identify patient sets of ILD development and the treatment status based on radiation reports and laboratory test results because identification using only recorded disease names may result in the misidentification of diagnosis and its development date. In the ILD Set, the CRP levels increased; however, in the ILD-GC Set, the CRP levels showed a more pronounced increase as the time window approached the onset of ILD, suggesting that the onset of ILD was more accurately captured. Furthermore, the overall incidence in the population was consistent with that observed in typical clinical settings. Hence, we mainly focused on the ILD-GC set, which is also important in severity.

Based on these results, we identified words with differences in word frequencies between the ILD-GC and No ILD Sets. As the words used varied depending on the writers (physicians or nurses), we organized words with the same meaning or background into terms before making comparisons. In the ILD-GC Set, within the time window immediately before the ILD onset date (days −1 to −30), there was a tendency for higher word frequencies of terms related to respiratory symptoms (Breathlessness, Shortness of breath, O_2_), Pain or Analgesics (Soreness, Ache, Pain, Pain-precordial, NRS, Lyrica [product name of pregabalin], Opioid, Oxinorm [product name of oxycodone], OxyContin [product name of oxycodone], OxyContin-increased, Oxycodone), and appetite (Inappetence, Food-intake, Feeling-queasy, Novamin [product name of prochlorperazine]).

The values of KL-6 and SP-D did not show abnormal levels (KL-6>500 U mL^−1^, SP-D>110 ng mL^−1^) or an increasing trend in the time window immediately before the ILD onset date (days –1 to −30). This can be interpreted to be caused by the same cut-off that was used for the abnormal values used in the patient-set identification algorithm by ILD development and treatment status. Hence, there was no impact on the diagnostic utility of KL-6 and SP-D. Additionally, there were many missing values in the test results, making it difficult to evaluate their trend. These missing values could be attributed to the fact that these tests were only performed when physicians suspected ILD and were not conducted in the absence of such suspicion. Some of the patients used anticancer drugs before the ILD onset date (days −1 to −30). PD-1 inhibitors, EGFR-TKIs, and ALK-TKIs are anticancer drugs that can cause ILD, which is consistent with previous reports [26,27]. In the ILD-GC Set, no words related to anticancer drug administration were identified among those with an increasing trend in the time window immediately before the ILD onset date (days −1 to −30). Words such as “reduction” and “discontinuation” were observed to have increased frequencies in physician documentation immediately before the ILD onset (d −30 to −1). Words such as “increase” and “administration” showed increased frequency in nursing documentation. However, owing to variations in anticancer drugs administered to different patients and insufficient sample size, it was not possible to identify definitive trends.

### Comparison With Prior Work

The word with the largest difference in the word frequency in the physicians’ notes was the brand name of pregabalin (Lyrica). Systematic reviews of pregabalin do not explicitly indicate a risk of ILD [47]. In contrast, an analysis using a spontaneous reporting database in Japan [48] reported a high odds ratio for interstitial pneumonia. Ethnic differences exist in the occurrence of drug-induced ILD; for example, gefitinib-induced ILD has a relatively high incidence in Japanese patients [49]. Although the absolute number of adverse events associated with pregabalin is small, there may be ethnic differences, making it easier to detect pregabalin as a risk factor than in other regions. Therefore, pregabalin may be considered a candidate risk factor for ILD. In hospitals, indirect factors for ILD onset can be discovered by examining the factors that lead to pregabalin use. Other neuropathic pain medications, such as mirogabalin, were not included in the analyzed data; therefore, they were not evaluated.

Poor performance status (PS) is known as a risk factor for interstitial pneumonia [50,51]. In our study, the PS score itself was not available from the dataset, and thus, we could not directly verify the relationship between the ILD onset and the poor PS score before the onset of ILD. On the other hand, the difference in word ratio for words included in respiratory symptom-related terms immediately before the onset date (days −1 to −30) was 170.59-46.51 in the physicians’ notes (170.59-46.51 more occurrences per 1000 articles compared to non-ILD cases) and 130.25-52.71 in the nurses’ notes.

Furthermore, among the words included in the pain- or analgesic-related terms, the difference in word ratio immediately before the onset date (days −1 to −30) was 209.76-45.16 in the physicians’ notes and 462.88-53.06 in the nurses’ notes. The group of terms associated with poor PS and palliative care consultation suggests local or systemic progression of lung cancer, which can be interpreted as approaching the end-of-life stage and considered consistent with the general clinical course.

There are reports that 72%‐83% of patients who actually received palliative care had a PS score of 2 or higher [52,53]. One reason for the lack of difference in the word frequency of PS itself may be that PS is an evaluation item, and the current word extraction method may not have been able to evaluate the actual score recording. Although PS is commonly used in clinical trials, respiratory symptom-related and pain- or analgesic-related terms indicate more specific patient conditions. We believe that future research can establish these as reliable risk factors for ILD, which can be useful for the prediction and early diagnosis of ILD in patients with stage IV lung cancer. Although respiratory symptoms and cancer pain change during the course of treatment, these symptoms are considered associated factors.

In the ILD-GC Set, respiratory symptom−related and appetite-related terms were more frequent immediately before the ILD onset date (days −1 to −30) compared to the No ILD Set, suggesting them as potential prodromal symptoms. Although respiratory symptom−related terms can be the initial symptoms of ILD, they may also indicate the disease progression of lung cancer itself. Rather than prodromal symptoms, they were considered associated factors.

Appetite-related words were documented more frequently by nurses. These were also considered more appropriate as risk factors rather than prodromal symptoms, as they likely reflect the deterioration of respiratory conditions or the side effects of anticancer drugs.

Delirium is known to be associated with the worsening of symptoms in patients living with cancer [54] or with the administration of anticancer drugs [55]. It can be considered an associated factor candidate, as it occurs more frequently in the period immediately preceding the ILD onset date (days −1 to −30) than in the No ILD Set. Moreover, sleepiness is thought to be a side effect of opioid administration (such as oxycodone and OxyContin) for cancer pain [56], and it indicates worsening of symptoms in patients with cancer. These can also be considered associated factors.

Although skin eruptions can be considered side effects of anticancer drugs [57], no words related to skin abnormalities other than “skin eruption,” such as rashes or skin, appeared. The fact that a rash appears alone suggests that there is no clear trend in skin abnormalities, and it may be a prodromal symptom or risk factor for ILD. However, there are limitations in its interpretation.

Both anemia and bleeding are known symptoms in patients with cancer [58,59] or side effects of anticancer drugs [59,60]. These words also represent the worsening of symptoms in patients with cancer. Since they are more frequent in the period immediately before ILD onset (days −1 to −30) compared to the No ILD Set, they can be considered associated factors.

Words such as “relief,” “decrease,” and “worsening” that supplement symptoms were not considered standalone words because they are used in combination with symptoms. “Residual” and “reduction” are considered to be words related to anticancer drug administration; however, they were excluded from consideration as standalone words because they could potentially be used in other contexts as well.

In this study, we were able to identify the risk factors for ILD onset, including a history of pregabalin use, respiratory symptom-related terms, and pain- or analgesic-related terms. On the other hand, we found that other terms (such as those that only express trends, eg, “relief,” “decrease,” or “worsening,” or those that only describe states, eg, “residual” or “reduction,” which are difficult to interpret as clear events on their own) did not reveal any noteworthy words. We suggest that there is a possibility of finding certain trends within free descriptions in EMRs, free-text narratives, and test result reports.

For words related to symptoms or medication names that showed increased word frequencies immediately before ILD onset (d −30 to −1) in either the ILD-GC Set or ILD Set, we confirmed an increase in the number of patients with these words documented, similar to the increase in word frequencies. Thus, the increased word frequency was not dependent on individual patients but rather the documentation rates of these words increased across multiple patients. This indicates that the words identified in this study may be candidates for prodromal symptoms or risk factors for ILD.

In this study, we analyzed free text from medical records using morphological analysis with Mecab and ComeJisyo. Because there is insufficient research on associated factors and prodromal symptoms of ILD onset, we began by analyzing traditional word frequencies to understand the actual situation. Although we identified words that had higher frequencies in the periods preceding ILD onset, we could not consider them with contextual interpretation. To resolve these limitations, we believe that in future studies, advanced LLMs could be a useful option for analyzing specific descriptions prior to onset with contextual interpretation.

### Limitations

This study used the medical records from a single institution. Moreover, information on precise diagnoses of ILD was not available, and patients with a history of ILD or concurrent ILD at the time of initial consultation were possibly included. However, our algorithms to identify patient sets of ILD seem to mitigate misidentification from the tendency of CRP levels to elevate in periods near the onset of ILD, especially in the ILD-GC Set. Furthermore, there may have been cases in which ILD developed after discharge; however, we were unable to conduct a verification that included such occurrences.

The evaluation was conducted based on the difference in frequencies of word occurrence in this study. This indicator was not based on previous studies but was devised and applied to identify symptoms or treatments. It should be noted that this indicator cannot truly determine whether symptoms actually occurred prior to ILD onset. Because this indicator is simply based on written records, we believe that it is useful for identifying words that appear with a specific frequency in populations or periods of interest. Further research is needed to determine the actual sensitivity of this approach. Moreover, negation words for events often follow nouns and verbs in Japanese sentences; thus, the terms may be extracted even from entries confirming the absence of symptoms. Thus, this is not the result of interpreting the context per se, even if the impact of the words routinely written from the standard rule for medical records or templates is mitigated by using the difference in word frequency. This study examined the possibility of extracting words that contribute to the prediction of ILD onset without contextual interpretation. Furthermore, the difference in frequency based on the term categories was not implemented owing to the difficulty of the comprehensive classification of the large amount of free text with medical concepts; nonetheless, the differences were observed at the word unit. Lastly, this study was not a confirmatory study with hypothesis testing but instead was an exploratory study. The interpretations from this study should be validated in further studies.

### Conclusions

In summary, we were able to identify respiratory symptom-related, pain- or analgesic-related, and appetite-related terms as associated factors for ILD using RWD from medical institutions providing acute treatment for malignant tumors. These results may be useful for the early detection of ILD in patients with stage IV lung cancer. We identified the potential of utilizing RWD to generate real-world evidence and its application in drug discovery and pharmaceutical development. The approach presented in this study suggests the possibility of identifying specific disease and risk factors leading to disease onset in a well-defined patient population.

## Supplementary material

10.2196/70603 Multimedia Appendix 1 The calculation formula and the results of supplementary analysis.

## Abbreviations

ALK-TKIs: anaplastic lymphoma kinase tyrosine kinase inhibitors
CRP: C-reactive protein
EGFR-TKIs: epidermal growth factor receptor tyrosine kinase inhibitors
EMR: electronic medical record
ILD: interstitial lung disease
ILD-GC Set: ILD with Glucocorticoid Treatment Set
KL-6: Krebs von den Lungen-6
LLMs: large language models
PD-1: programmed cell death protein 1
PS: performance status
RWD: real-world data
SP-D: surfactant protein D
