# Supplementary material for: Identifying Terminologies Used Prior to the Onset of Interstitial Lung Disease in Patients With Lung Cancer: Descriptive Analysis of Electronic Medical Record Data
Source: JMIR Cancer. 2025 Nov 3;11:e70603. doi: 10.2196/70603 (PMC12582523; doi:10.2196/70603)
Supplement: Multimedia Appendix 1 [file cancer-v11-e70603-s001.docx]

## Multimedia Appendix 1

**Supplementary Material 1. The formula for calculation of difference of frequency of word in -30 to -1 day(s) between the ILD-GC and No-ILD Sets**

$$\text{(1000 × }N_{ILD-GC}\text{ / }A_{ILD-GC}\text{)}-\text{(1000 × }N_{No-ILD}\text{ / }A_{No-ILD}\text{)}$$

$N_{ILD-GC}$ : Word appearance count in the ILD-GC Set 1–30 days before ILD onset in the free text of medical records.
$A_{ILD-GC}$ : Total number of medical records in the ILD-GC Set 1–30 days before ILD onset in the free text of medical records.

$N_{No-ILD}$ : Word appearance count in the No-ILD Set in the free text of medical records.
$A_{No-ILD}$ : Total number of medical records in the No-ILD Set in the free text of medical records.

**Supplementary Table 1. The difference of frequency of word written by physicians between ILD Set and No ILD Set**

| Word | Difference in the frequency of words in -30 to -1 day(s) | Number of patients for which the words were recorded in -30 to -1 day(s) | Difference in the frequency of words in -31 to -60 day(s) | Difference in the frequency of words in -61 to -90 day(s) | Term Category |
| --- | --- | --- | --- | --- | --- |
|  |  |  |  |  |  |
| **O2** | 46.12 | 52 | 27.12 | -0.50 | Respiratory symptom-related |
| **Sleepiness** | 44.62 | 9 | 11.20 | -13.66 | Sleepiness |
| **Fever** | 42.87 | 31 | 17.68 | 30.05 |  |
| **Soreness** | 38.63 | 20 | 56.78 | -26.64 | Pain/ analgesic-related |
| **Oxycodone** | 37.73 | 9 | -2.22 | -3.06 | Pain/ analgesic-related |
| **RA** | 37.40 | 22 | 46.86 | 9.43 |  |
| **Sp** | 36.39 | 53 | 26.07 | 18.91 |  |
| **Improvement** | 36.12 | 26 | 10.06 | 29.13 |  |
| **Tendency-to-improve** | 36.11 | 19 | 8.67 | -4.68 |  |
| **HR** | 34.28 | 31 | 41.48 | -10.01 |  |
| **Breathlessness** | 33.84 | 10 | -0.21 | -9.29 | Respiratory symptom-related |
| **Restart** | 33.19 | 9 | 24.82 | -5.27 |  |
| **Lyrica (product name of pregabalin)** | 32.99 | 5 | -2.57 | -12.46 | Pain/ Analgesic-Related |
| **BP** | 32.44 | 35 | 36.03 | -20.01 |  |
| **RT** | 32.43 | 14 | 36.91 | 0.96 |  |
| **Tenderness** | 32.22 | 11 | 4.35 | -6.24 |  |
| **Morning** | 32.21 | 19 | 8.54 | -0.43 |  |
| **NRS** | 31.42 | 11 | -15.74 | -19.87 | Pain/ analgesic-related |
| **Anemia** | 30.83 | 9 | 3.78 | -2.97 | Anemia |
| **Use** | 29.30 | 16 | 5.50 | -12.65 |  |
| **Opso (product name of morphine)** | 29.08 | 4 | -4.51 | -1.23 |  |
| **ERCP** | 28.86 | 2 | -0.28 | -0.28 |  |
| **Worsening** | 28.35 | 22 | 11.57 | -1.95 |  |
| **Yesterday** | 28.10 | 28 | 29.50 | 21.10 |  |
| **Stent** | 28.00 | 3 | 0.24 | -1.14 |  |
| **Cause** | 27.53 | 13 | 3.02 | 6.55 |  |
| **Pain** | 26.07 | 31 | 34.43 | -46.19 | Pain/ analgesic-related |
| **Olanzapine** | 25.47 | 3 | 6.08 | -1.34 |  |
| **Stomatitis** | 25.37 | 8 | 6.90 | 4.39 |  |
| **Night** | 25.37 | 16 | 16.93 | -4.93 |  |
| **Oral-administration** | 25.16 | 23 | 6.05 | -12.13 |  |
| **Metastasis** | 24.83 | 22 | 49.90 | -25.28 |  |
| **Impact** | 24.54 | 19 | 4.91 | -6.92 |  |
| **BT** | 23.57 | 27 | 55.92 | -30.04 |  |
| **Exacerbation** | 23.53 | 17 | -13.59 | -9.11 |  |
| **Continuation** | 23.37 | 23 | 26.51 | 4.72 |  |
| **Weight** | 23.34 | 20 | 17.51 | 7.02 |  |
| **Oxyfast (product name of oxycodone)** | 23.16 | 2 | 13.80 | 1.01 |  |
| **Myoclonus** | 23.03 | 2 | 13.32 | -0.28 |  |
| **Place / When-One-did-that (noun / conjunctive particle word in Japanese)** | 22.91 | 17 | 12.42 | -5.06 |  |
| **Delirium** | 22.70 | 5 | 6.56 | -16.93 | Delirium |
| **Implementation** | 21.98 | 13 | 10.04 | -0.17 |  |
| **Opioid** | 21.82 | 8 | 6.42 | -7.32 | Pain/ analgesic-related |
| **Rescue** | 21.67 | 8 | -1.75 | -20.29 |  |
| **This-day** | 21.42 | 32 | 28.55 | 34.24 |  |
| **Meal** | 21.13 | 19 | 11.77 | 21.13 |  |
| **Right-hypochondrium** | 20.60 | 2 | -0.30 | -1.54 |  |
| **Drainage** | 20.53 | 9 | 21.63 | 7.71 |  |
| **Current** | 20.03 | 19 | 5.83 | -10.27 |  |
| **Today** | 19.83 | 15 | 3.51 | -18.63 |  |

**Supplementary Table 2. The difference of frequency of word written by nurses between ILD Set and No ILD Set**

| Word | Difference in the frequency of words in -30 to -1 day(s) | Number of patients for which the words were recorded in -30 to -1 day(s) | Difference in the frequency of words in -31 to -60 day(s) | Difference in the frequency of words in -61 to -90 day(s) | Term Category |
| --- | --- | --- | --- | --- | --- |
|  |  |  |  |  |  |
| **Use** | 79.32 | 36 | 58.23 | -73.48 |  |
| **Oral-administration** | 76.86 | 33 | 52.98 | 17.33 |  |
| **Oxycodone** | 64.13 | 8 | -14.46 | -10.76 | Pain/ analgesic-related |
| **OxyContin (product name of oxycodone)** | 62.91 | 8 | 43.34 | -41.61 | Pain/ analgesic-related |
| **Opioid** | 53.82 | 14 | 1.82 | 12.47 | Pain/ analgesic-related |
| **Soreness** | 52.41 | 32 | -33.13 | -146.55 | Pain/ analgesic-related |
| **Stomatitis** | 52.37 | 4 | 1.81 | -8.61 |  |
| **Breathlessness** | 49.02 | 15 | -12.21 | -33.28 | Respiratory symptom-related |
| **Daytime** | 47.96 | 17 | 4.94 | -25.61 |  |
| **Course** | 47.26 | 26 | 32.78 | 61.72 |  |
| **During movement** | 46.11 | 14 | 11.47 | -39.69 |  |
| **NSCLC** | 45.99 | 9 | 1.95 | -4.53 |  |
| **Fever** | 44.87 | 11 | 15.49 | 0.90 |  |
| **Fall-risk** | 43.45 | 28 | 39.39 | 28.28 |  |
| **Hgb** | 43.25 | 27 | 4.89 | 39.84 |  |
| **Everyday** | 42.42 | 12 | -6.87 | -5.48 |  |
| **On-bed** | 41.09 | 11 | 20.85 | -23.37 |  |
| **Hospitalization** | 39.28 | 33 | -45.15 | 69.43 |  |
| **Novamin (Product name of Prochlorperazine)** | 37.10 | 8 | 14.31 | 5.75 | Appetite-related |
| **Sleepiness** | 36.94 | 10 | 20.51 | -17.92 | Sleepiness |
| **Gilotrif (product name of afatinib)** | 35.78 | 2 | 13.93 | -6.90 |  |
| **Okay** | 34.83 | 27 | 22.98 | 3.53 |  |
| **Discontinuation** | 34.58 | 12 | -9.04 | -4.18 |  |
| **Increase** | 34.03 | 7 | 13.79 | -8.20 |  |
| **NRS** | 33.20 | 16 | -12.19 | -90.90 | Pain/ analgesic-related |
| **Loxoprofen** | 32.47 | 10 | 45.26 | 2.44 |  |
| **Nurse-call** | 31.18 | 10 | -7.27 | -34.58 |  |
| **Albumin** | 30.83 | 14 | -22.01 | -11.83 |  |
| **Redness** | 30.76 | 14 | 15.18 | 14.26 |  |
| **Dryness** | 30.47 | 12 | -3.15 | -5.24 |  |
| **Edema** | 29.49 | 7 | 1.97 | -22.34 |  |
| **RA** | 29.31 | 8 | 10.51 | -2.91 |  |
| **Marked** | 28.89 | 9 | 8.73 | -18.58 |  |
| **Administration** | 28.87 | 16 | 5.58 | -1.60 |  |
| **Response** | 28.46 | 9 | -2.62 | -25.54 |  |
| **Pneumonia** | 27.74 | 4 | -5.38 | -8.85 |  |
| **Fall** | 27.31 | 34 | 10.12 | 114.98 |  |
| **Collection-date** | 27.27 | 32 | -0.76 | 73.54 |  |
| **Transfusion** | 26.88 | 5 | -5.81 | -12.75 |  |
| **At-rest** | 26.82 | 8 | -17.22 | -16.30 |  |
| **Right-precordial** | 26.35 | 4 | 16.69 | -4.14 |  |
| **CBDCA** | 25.99 | 10 | 27.43 | -7.53 |  |
| **Purpose** | 25.95 | 20 | 13.93 | -3.20 |  |
| **Shortness of breath** | 25.22 | 12 | 45.04 | 26.98 | Respiratory symptom-related |
| **Implementation** | 24.88 | 17 | 11.16 | -6.90 |  |
| **Dyspnea** | 24.56 | 5 | 11.43 | -5.93 |  |
| **Introduction** | 24.25 | 9 | -7.17 | -9.72 |  |
| **During-walking** | 24.08 | 24 | 48.81 | 58.99 |  |
| **Dizziness / Vertigo** | 24.06 | 20 | -6.68 | 9.29 |  |
| **Unsteady-walking** | 23.83 | 20 | 18.75 | 46.53 |  |

**Supplementary Table 3. The number of patients for which the words were recorded by physicians in the ILD GC Set**

| Word | Number of patients for which the words were recorded in -30 to -1 day(s) | Number of patients for which the words were recorded in -60 to -31 day(s) | Number of patients for which the words were recorded in -90 to -61 day(s) | Term Category |
| --- | --- | --- | --- | --- |
|  |  |  |  |  |
| **Lyrica (product name of pregabalin)** | 3 | 3 | 1 | Pain/Analgesic-Related |
| **Pain** | 8 | 6 | 3 | Pain/Analgesic-Related |
| **Sleepiness** | 2 | 2 | 0 | Sleepiness |
| **Breathlessness** | 2 | 3 | 2 | Respiratory symptom-related |
| **Yesterday** | 8 | 6 | 4 |  |
| **Improvement** | 8 | 6 | 4 |  |
| **Morning** | 5 | 4 | 4 |  |
| **AM** | 4 | 4 | 2 |  |
| **Soreness** | 7 | 5 | 2 | Pain/Analgesic-Related |
| **Restart** | 5 | 3 | 0 |  |
| **Reduction** | 3 | 2 | 0 |  |
| **Degree** | 4 | 3 | 1 |  |
| **After** | 6 | 5 | 2 |  |
| **Worsening** | 5 | 5 | 2 |  |
| **Meal** | 5 | 4 | 3 |  |
| **Anxiety** | 2 | 1 | 0 |  |
| **NRS** | 4 | 1 | 1 | Pain/Analgesic-Related |
| **Anemia** | 2 | 1 | 0 | Anemia |
| **This-day** | 6 | 6 | 3 |  |
| **Today** | 4 | 3 | 2 |  |
| **Oxycodone** | 3 | 2 | 1 | Pain/Analgesic-Related |
| **Tomorrow** | 5 | 4 | 2 |  |
| **Okay** | 3 | 4 | 3 |  |
| **Night** | 2 | 4 | 2 |  |
| **Right-chest** | 4 | 2 | 2 |  |
| **Decrease** | 6 | 5 | 1 |  |
| **Exacerbation** | 3 | 4 | 1 |  |
| **Delirium** | 2 | 1 | 0 | Delirium |
| **Bleeding** | 2 | 1 | 0 | Bleeding |
| **Discontinuation** | 3 | 7 | 2 |  |
| **Bleeding-source** | 2 | 0 | 0 |  |
| **Skin-eruption** | 3 | 1 | 2 | Skin-eruption |
| **Increase** | 3 | 4 | 2 |  |
| **Use** | 5 | 2 | 1 |  |
| **Test / Trial** | 2 | 1 | 0 |  |
| **Condition** | 3 | 3 | 2 |  |
| **Cause** | 3 | 3 | 2 |  |
| **Response** | 3 | 3 | 2 |  |
| **Ache** | 3 | 3 | 1 | Pain/ analgesic-related |
| **O_2_** | 7 | 8 | 6 | Respiratory symptom-related |
| **Consideration** | 7 | 7 | 3 |  |
| **Conversation** | 2 | 2 | 1 |  |
| **Last night** | 3 | 2 | 0 |  |
| **Rescue** | 2 | 3 | 1 |  |
| **OxyContin (product name of oxycodone)** | 3 | 1 | 1 | Pain/ analgesic-related |
| **Awareness** | 3 | 3 | 1 |  |
| **Nasal** | 2 | 1 | 0 |  |
| **Face** | 2 | 1 | 1 |  |
| **Drain** | 2 | 2 | 0 |  |
| **Residual** | 4 | 3 | 1 |  |

**Supplementary Table 4. The number of patients for which the words were recorded by nurses in the ILD GC Set**

| Word | Number of patients for which the words were recorded in -30 to -1 day(s) | Number of patients for which the words were recorded in -60 to -31 day(s) | Number of patients for which the words were recorded in -90 to -61 day(s) | Term Category |
| --- | --- | --- | --- | --- |
|  |  |  |  |  |
| **Pain** | 7 | 5 | 2 | Pain/Analgesic-Related |
| **OxyContin (product name of oxycodone)** | 4 | 2 | 2 | Pain/Analgesic-Related |
| **NRS** | 7 | 3 | 4 | Pain/Analgesic-Related |
| **Oxinorm (product name of oxycodone)** | 5 | 2 | 3 | Pain/Analgesic-Related |
| **Oral-administration** | 7 | 7 | 4 |  |
| **Impact** | 5 | 4 | 1 |  |
| **Opioid** | 5 | 3 | 3 | Pain/Analgesic-Related |
| **Most** | 3 | 2 | 1 |  |
| **Worry** | 2 | 1 | 0 |  |
| **Breathlessness** | 4 | 5 | 1 | Respiratory symptom-related |
| **Increase** | 3 | 2 | 1 |  |
| **Course** | 6 | 5 | 4 |  |
| **This-day** | 9 | 5 | 2 |  |
| **Inappetence** | 3 | 1 | 0 | Appetite-related |
| **Soreness** | 8 | 5 | 4 | Pain/Analgesic-Related |
| **Drug-name** | 2 | 1 | 0 |  |
| **Usage** | 2 | 1 | 1 |  |
| **Right-back** | 3 | 1 | 1 |  |
| **AM** | 2 | 2 | 2 |  |
| **Possibility** | 4 | 1 | 0 |  |
| **Administration** | 3 | 2 | 0 |  |
| **Anxiety** | 3 | 2 | 0 |  |
| **During movement** | 4 | 2 | 0 |  |
| **Novamin (product name of prochlorperazine)** | 3 | 1 | 1 | Appetite-related |
| **Family** | 2 | 1 | 1 |  |
| **Timing** | 2 | 0 | 1 |  |
| **Shortness of breath** | 3 | 2 | 1 | Respiratory symptom-related |
| **Fall** | 6 | 6 | 5 |  |
| **Doctor** | 4 | 3 | 2 |  |
| **Start** | 4 | 2 | 1 |  |
| **Watchful-waiting** | 4 | 0 | 1 |  |
| **OxyContin-increased** | 2 | 0 | 0 | Pain/Analgesic-related |
| **Face-scale** | 2 | 1 | 1 |  |
| **Pattern** | 2 | 1 | 1 |  |
| **Relief** | 4 | 4 | 2 |  |
| **Pain-precordial** | 3 | 1 | 1 | Pain/ analgesic-related |
| **Fall-risk** | 6 | 5 | 3 |  |
| **Going-out** | 2 | 2 | 2 |  |
| **Today** | 4 | 3 | 0 |  |
| **Medical-condition** | 2 | 3 | 1 |  |
| **Feeling-queasy** | 2 | 2 | 2 | Appetite-related |
| **Before** | 3 | 3 | 5 |  |
| **Sp** | 3 | 4 | 2 |  |
| **Hgb** | 3 | 5 | 0 |  |
| **Oxycodone** | 2 | 2 | 1 | Pain/ analgesic-related |
| **O_2_** | 3 | 4 | 2 | Respiratory symptom-related |
| **Food-intake** | 3 | 0 | 0 | Appetite-related |
| **Intermittent** | 2 | 1 | 1 |  |
| **Discontinuation** | 2 | 2 | 1 |  |
| **Average** | 2 | 0 | 1 |  |

**Supplementary Table 5. The number of patients for which the words were recorded by physicians in the ILD Set**

| Word | Number of patients for which the words were recorded in -30 to -1 day(s) | Number of patients for which the words were recorded in -60 to -31 day(s) | Number of patients for which the words were recorded in -90 to -61 day(s) | Term Category |
| --- | --- | --- | --- | --- |
|  |  |  |  |  |
| **O2** | 52 | 49 | 31 | Respiratory symptom-related |
| **Sleepiness** | 9 | 7 | 1 | Sleepiness |
| **Fever** | 31 | 27 | 14 |  |
| **Soreness** | 20 | 18 | 5 | Pain/ analgesic-related |
| **Oxycodone** | 9 | 6 | 1 | Pain/ analgesic-related |
| **RA** | 22 | 18 | 8 |  |
| **Sp** | 53 | 49 | 32 |  |
| **Improvement** | 26 | 33 | 17 |  |
| **Tendency-to-improve** | 19 | 14 | 5 |  |
| **HR** | 31 | 34 | 15 |  |
| **Breathlessness** | 10 | 7 | 3 | Respiratory symptom-related |
| **Restart** | 9 | 7 | 3 |  |
| **Lyrica (product name of pregabalin)** | 5 | 3 | 0 | Pain/ analgesic-related |
| **BP** | 35 | 26 | 11 |  |
| **RT** | 14 | 11 | 6 |  |
| **Tenderness** | 11 | 12 | 5 |  |
| **Morning** | 19 | 12 | 6 |  |
| **NRS** | 11 | 6 | 2 | Pain/ analgesic-related |
| **Anemia** | 9 | 7 | 4 | Anemia |
| **Use** | 16 | 10 | 2 |  |
| **Opso (product name of morphine)** | 4 | 3 | 2 |  |
| **ERCP** | 2 | 0 | 0 |  |
| **Worsening** | 22 | 22 | 12 |  |
| **Yesterday** | 28 | 27 | 15 |  |
| **Stent** | 3 | 2 | 1 |  |
| **Cause** | 13 | 9 | 5 |  |
| **Pain** | 31 | 32 | 16 | Pain/ analgesic-related |
| **Olanzapine** | 3 | 2 | 0 |  |
| **Stomatitis** | 8 | 10 | 6 |  |
| **Night** | 16 | 14 | 4 |  |
| **Oral-administration** | 23 | 19 | 13 |  |
| **Metastasis** | 22 | 26 | 9 |  |
| **Impact** | 19 | 12 | 5 |  |
| **BT** | 27 | 21 | 9 |  |
| **Exacerbation** | 17 | 12 | 8 |  |
| **Continuation** | 23 | 23 | 14 |  |
| **Weight** | 20 | 20 | 9 |  |
| **Oxyfast (product name of oxycodone)** | 2 | 2 | 1 |  |
| **Myoclonus** | 2 | 1 | 0 |  |
| **Place / When-One-did-that (noun / conjunctive particle word in Japanese)** | 17 | 19 | 10 |  |
| **Delirium** | 5 | 3 | 0 | Delirium |
| **Implementation** | 13 | 15 | 3 |  |
| **Opioid** | 8 | 4 | 1 | Pain/ analgesic-related |
| **Rescue** | 8 | 4 | 0 |  |
| **This-day** | 32 | 36 | 17 |  |
| **Meal** | 19 | 13 | 12 |  |
| **Right-hypochondrium** | 2 | 1 | 0 |  |
| **Drainage** | 9 | 10 | 3 |  |
| **Current** | 19 | 14 | 7 |  |
| **Today** | 15 | 19 | 7 |  |

**Supplementary Table 6. The number of patients for which the words were recorded by nurses in the ILD Set**

| Word | Number of patients for which the words were recorded in -30 to -1 day(s) | Number of patients for which the words were recorded in -60 to -31 day(s) | Number of patients for which the words were recorded in -90 to -61 day(s) | Term Category |
| --- | --- | --- | --- | --- |
|  |  |  |  |  |
| **Use** | 36 | 24 | 11 |  |
| **Oral-administration** | 33 | 29 | 17 |  |
| **Oxycodone** | 8 | 7 | 3 | Pain/ analgesic-related |
| **OxyContin (product name of oxycodone)** | 8 | 7 | 4 | Pain/ analgesic-related |
| **Opioid** | 14 | 6 | 5 | Pain/ analgesic-related |
| **Soreness** | 32 | 26 | 13 | Pain/ analgesic-related |
| **Stomatitis** | 4 | 2 | 0 |  |
| **Breathlessness** | 15 | 11 | 4 | Respiratory symptom-related |
| **Daytime** | 17 | 13 | 5 |  |
| **Course** | 26 | 20 | 13 |  |
| **During movement** | 14 | 8 | 2 |  |
| **NSCLC** | 9 | 3 | 1 |  |
| **Fever** | 11 | 8 | 5 |  |
| **Fall-risk** | 28 | 27 | 16 |  |
| **Hgb** | 27 | 24 | 14 |  |
| **Everyday** | 12 | 4 | 2 |  |
| **On-bed** | 11 | 4 | 2 |  |
| **Hospitalization** | 33 | 21 | 18 |  |
| **Novamin (Product name of Prochlorperazine)** | 8 | 3 | 3 | Appetite-related |
| **Sleepiness** | 10 | 8 | 4 | Sleepiness |
| **Gilotrif (product name of afatinib)** | 2 | 2 | 0 |  |
| **Okay** | 27 | 25 | 10 |  |
| **Discontinuation** | 12 | 4 | 2 |  |
| **Increase** | 7 | 7 | 1 |  |
| **NRS** | 16 | 12 | 7 | Pain/ analgesic-related |
| **Loxoprofen** | 10 | 8 | 3 |  |
| **Nurse-call** | 10 | 4 | 1 |  |
| **Albumin** | 14 | 10 | 6 |  |
| **Redness** | 14 | 8 | 6 |  |
| **Dryness** | 12 | 5 | 3 |  |
| **Edema** | 7 | 5 | 0 |  |
| **RA** | 8 | 6 | 1 |  |
| **Marked** | 9 | 2 | 1 |  |
| **Administration** | 16 | 10 | 6 |  |
| **Response** | 9 | 8 | 3 |  |
| **Pneumonia** | 4 | 1 | 0 |  |
| **Fall** | 34 | 22 | 21 |  |
| **Collection-date** | 32 | 29 | 20 |  |
| **Transfusion** | 5 | 1 | 0 |  |
| **At-rest** | 8 | 3 | 2 |  |
| **Right-precordial** | 4 | 4 | 0 |  |
| **CBDCA** | 10 | 10 | 3 |  |
| **Purpose** | 20 | 19 | 6 |  |
| **Shortness of breath** | 12 | 10 | 4 | Respiratory symptom-related |
| **Implementation** | 17 | 11 | 3 |  |
| **Dyspnea** | 5 | 3 | 0 |  |
| **Introduction** | 9 | 2 | 1 |  |
| **During-walking** | 24 | 20 | 14 |  |
| **Dizziness / Vertigo** | 20 | 15 | 10 |  |
| **Unsteady-walking** | 20 | 14 | 9 |  |

**Supplementary Table 7. Correspondence table of original terms used in Japanese medical charts and their English translations**

| English translations | Original Japanese terms |
| --- | --- |
|  |  |
| **Administration** | 投与 |
| **After** | 後 |
| **Albumin** | アルブミン |
| **AM** | AM |
| **Anemia** | 貧血 |
| **Anxiety** | 不安 |
| **At-rest** | 安静時 |
| **Average** | 平均 |
| **Awareness** | 自覚 |
| **Before** | 前 |
| **Bleeding** | 出血 |
| **Bleeding-source** | 出血源 |
| **BP** | BP |
| **BT** | BT |
| **Cause** | 原因 |
| **CBDCA** | CBDCA |
| **Collection-date** | 採取日 |
| **Condition** | 調子 |
| **Medical-condition** | 病状 |
| **Consideration** | 検討 |
| **Continuation** | 継続 |
| **Conversation** | 会話 |
| **Course** | 経過 |
| **Current** | 現在 |
| **Daytime** | 日中 |
| **Decrease** | 低下 |
| **Degree** | 程度 |
| **Delirium** | せん妄 |
| **Discontinuation** | 中止 |
| **Dizziness / Vertigo** | めまい |
| **Doctor** | 先生 |
| **Drain** | ドレーン |
| **Drainage** | 排液 |
| **Drug-name** | 薬剤名 |
| **Dryness** | 乾燥 |
| **During movement** | 体動時 |
| **During-walking** | 歩行時 |
| **Breathlessness** | 呼吸苦 |
| **Dyspnea** | 呼吸困難 |
| **Edema** | 浮腫 |
| **ERCP** | ERCP |
| **Everyday** | 毎日 |
| **Exacerbation** | 増悪 |
| **Face** | 顔面 |
| **Face-scale** | フェイススケール |
| **Fall** | 転倒 |
| **Fall-risk** | 転倒リスク |
| **Family** | 家族 |
| **Feeling-queasy** | 吐き気 |
| **Fever** | 発熱 |
| **Food-intake** | 食事摂取量 |
| **Gilotrif (product name of afatinib)** | ジオトリフ |
| **Going-out** | 外出 |
| **Hgb** | Hgb |
| **Hospitalization** | 入院 |
| **HR** | HR |
| **Impact** | 影響 |
| **Implementation** | 施行 |
| **Improvement** | 改善 |
| **Inappetence** | 食欲不振 |
| **Increase** | 増量 |
| **Intermittent** | 間欠的 |
| **Introduction** | 導入 |
| **Last night** | 昨夜 |
| **Loxoprofen** | ロキソプロフェン |
| **Lyrica (product name of pregabalin)** | リリカ |
| **Marked** | 著明 |
| **Meal** | 食事 |
| **Metastasis** | 転移 |
| **Morning** | 朝 |
| **Most** | 一番 |
| **Myoclonus** | ミオクローヌス |
| **nasal** | nasal |
| **Night** | 夜 |
| **Novamin (product name of prochlorperazine)** | ノバミン |
| **NRS** | NRS |
| **NSCLC** | NSCLC |
| **Nurse-call** | ナースコール |
| **O2** | O2 |
| **Okay** | 大丈夫 |
| **Olanzapine** | オランザピン |
| **On-bed** | ベッド上 |
| **Opioid** | オピオイド |
| **Opso (product name of morphine)** | オプソ |
| **Oral-administration** | 内服 |
| **Oxinorm (product name of oxycodone)** | オキノーム |
| **Oxycodone** | オキシコドン |
| **OxyContin (product name of oxycodone)** | オキシコンチン |
| **OxyContin-increased** | オキシコンチン増量 |
| **Oxyfast (product name of oxycodone)** | オキファスト |
| **Ache** | 痛 |
| **Pain** | 痛み |
| **Soreness** | 疼痛 |
| **Pain-precordial** | 前胸部痛 |
| **Pattern** | パターン |
| **Place / When-One-did-that (noun / conjunctive particle word in Japanese)** | ところ |
| **Pneumonia** | 肺炎 |
| **Possibility** | 可能性 |
| **Purpose** | 目的 |
| **RA** | RA |
| **Redness** | 発赤 |
| **Relief** | 軽減 |
| **Reduction** | 減量 |
| **Rescue** | レスキュー |
| **Residual** | 残存 |
| **Response** | 対応 |
| **Restart** | 再開 |
| **Right-back** | 右背部 |
| **Right-chest** | 右胸 |
| **Right-hypochondrium** | 右季肋部 |
| **Right-precordial** | 右前胸部 |
| **RT** | RT |
| **Shortness of breath** | 息切れ |
| **Skin-eruption** | 皮疹 |
| **Sleepiness** | 眠気 |
| **Sp** | Sp |
| **Start** | 開始 |
| **Stent** | ステント |
| **Stomatitis** | 口内炎 |
| **Tendency-to-improve** | 改善傾向 |
| **Tenderness** | 圧痛 |
| **Test / Trial** | 試験 |
| **Timing** | 時期 |
| **Today** | 今日 |
| **This-day** | 本日 |
| **Tomorrow** | 明日 |
| **Transfusion** | 輸血 |
| **Unsteady-walking** | 歩行時ふらつき |
| **Usage** | 使用量 |
| **Use** | 使用 |
| **Watchful-waiting** | 経過観察 |
| **Weight** | 体重 |
| **Worry** | 心配 |
| **Worsening** | 悪化 |
| **Yesterday** | 昨日 |
